# Reporting Down syndrome on the death certificate for Alzheimer disease/unspecified dementia deaths

**DOI:** 10.1371/journal.pone.0281763

**Published:** 2023-02-13

**Authors:** Scott D. Landes, Julia M. Finan, Margaret A. Turk

**Affiliations:** 1 Department of Sociology and Aging Studies Institute, Maxwell School of Citizenship and Public Affairs, Syracuse University, Syracuse, NY, United States of America; 2 Department of Physical Medicine & Rehabilitation, SUNY Upstate Medical University, Syracuse, NY, United States of America; Institut du cerveau et de la moelle épinière: Institut du cerveau et de la moelle epiniere, FRANCE

## Abstract

**Background:**

Death certificates are crucial for understanding population health trends including the burden of disease mortality. Accurate reporting of causes of death on these records is necessary in order to implement adequate public health policies and fund disease research. While there is evidence that Alzheimer disease and unspecified dementia are prevalent among people with Down syndrome, a 2014 Centers for Disease Control and Prevention (CDC) rule change instructing that Down syndrome should be reported as the underlying cause of death in instances when death occurred from Alzheimer disease or unspecified dementia threatens the accuracy and the utility of death certificates for this population.

**Methods:**

This study used 15 years (2005–2019) of US death certificate data for adults with and without Down syndrome. We compare the mortality burden due to Alzheimer disease and unspecified dementia prior to and after amending death certificates that report Down syndrome as the underlying cause of death.

**Results:**

When analyzing death certificates without addressing the reporting of Down syndrome as the underlying cause of death, rates of death due to Alzheimer disease and dementia ranked as the third leading cause of death for both adults with and without Down syndrome. After amending death certificates that reported Down syndrome as the underlying cause of death, Alzheimer disease and dementia were the leading cause of death among those with Down syndrome, occurring 2.7 times more in adults with compared to without Down syndrome.

**Conclusion:**

The findings of this study highlight the importance of accurate mortality data for studying and addressing population health trends. The current practice of reporting Down syndrome as the underlying cause of death rather than the disease responsible for death needs to be reconsidered and modified. If not, people with Down syndrome may be further marginalized within dementia related support and research.

## Background

Death certificates are a critical data source for monitoring population health [1–4]. An example of the US Standard Certificate of Death can be found at the Centers for Disease Control and Prevention (CDC) website [5]. Though death certificates include demographic information, place of death, and a list of multiple comorbidities present at the time of death, population health strategies are primarily informed by analysis of the underlying cause of death [6], the illness or injury that started the chain of events leading to death [7]. In the US, the underlying cause of death is the data point that is used to tabulate the leading causes of death reported by the CDC [8], giving insight into the “burden” of particular disease mortality on the population [8–11]. Accurate evidence on the burden of disease mortality is vital for developing effective public health policies aimed at preventing premature mortality and adequately addressing the health needs of the population [12]. This is especially pertinent for Alzheimer disease due to evidence that the number of deaths from this disease has more than doubled over the past 20 years [13, 14]. Pre-pandemic, Alzheimer disease was the 6^th^ leading cause of death in the US [15, 16]. However, it is important to recognize that when combined, Alzheimer disease and unspecified dementia ranked as the 3^rd^ leading cause of death in the US [17, 18].

Despite the importance of the underlying cause of death, the reporting of this illness or injury is not always straightforward [2, 19]. It is the clinical opinion of the physician [7, 20, 21] or other medical personnel [22] that is responsible for certifying the medical portion of the death certificate. The certifier is often required to delineate between multiple comorbidities that were present at the time of death [23–25]. Inadequate training in death certification that medical personnel typically receive [23, 26] in combination with frequent death certification by clinicians who had no prior care responsibilities for the decedents [19, 25] contribute to an often high degree of uncertainty surrounding the accuracy of this process [27]. In addition, states have varying regulations on who is permitted to act as a death certifier, resulting in inconsistent backgrounds and knowledge regarding the death certification process [22, 28].

The death certification process becomes even more complex for intellectually and developmentally disabled people. Intellectually and developmentally disabled people are a marginalized health population experiencing multiple health disparities and barriers to healthcare [29–31]. Beyond concerns regarding access to care [30], even when receiving care, medical personnel typically do not have adequate training or knowledge of the health needs of disabled people [30, 32–37]. In turn, they may not be aware of common causes of death among this population and/or may hold stereotypes that inaccurately conflate disability and health [37–40]. This often leads to postmortem diagnostic overshadowing, when intellectual or developmental disabilities, which are disabilities not illnesses, are reported as the underlying cause of death [41–45]. When cause of death trends are ‘obscured’ by postmortem diagnostic overshadowing, the usefulness of underlying cause of death data is compromised.

One particular instance of concern is the reporting of Alzheimer disease and unspecified dementia on the death certificates of people with Down syndrome. The need for accurate information on the burden of Alzheimer related disease mortality is even more pressing for adults with Down syndrome given the high prevalence of this disease within this population. It is estimated that 11.3% of those in the general population over the age of 65 have Alzheimer disease [46]. In contrast, according to the National Down Syndrome Society, about half of the population of individuals with Down syndrome have Alzheimer disease by the age of 60 [47]. Furthermore, there is evidence that nearly all adults with Down syndrome develop Alzheimer disease neuropathology by 40 years of age [48]. Due primarily to improvements in the medical care system, the average age of death for those with Down syndrome increased from 12 years in 1949 to around 60 years per studies using data from 2008 and 2017 [49, 50]. With this rise in age of death among people with Down syndrome, it is reasonable to think that the prevalence of Alzheimer related dementia is likely to increase as dementia risk for this population is documented as increasing from 23% among those age 50 to 80% among those aged 65 years and above [51].

Beyond the general challenges presented by insufficient training in death certification and/or lack of medical personnel knowledge regarding Down syndrome, instructions on reporting the underlying cause of death for people with Down syndrome were modified in 2013. In 2011, the WHO approved a proposal which changed the selection rules for reporting the underlying cause of death in instances when Alzheimer disease or unspecified dementia were the cause of death for people with Down syndrome. The modification added the following: “Unspecified dementia (F03) and Alzheimer’s disease (G30.-) should be considered an obvious consequence of Down’s syndrome (Q90.-)” [52]. This change to the guidelines was implemented in 2013 [52], established as official record in the CDC’s instruction manuals guiding death certification in the US in 2014 [53], and incorporated into the automated system that the National Center for Health Statistics (NCHS) uses to correct death certificate errors [54]. Thus, either through changing the practice of medical personnel certifying death certificates, or correcting errors via the automated system, the new selection rule necessitates that when an individual with Down syndrome dies from Alzheimer disease or unspecified dementia that the underlying cause of death is not reported as Alzheimer disease or unspecified dementia, but as Down syndrome.

This change is problematic. Individuals with Down syndrome are an overlooked population who die at younger ages than the general population [49, 50], have a comparatively higher prevalence of Alzheimer disease or unspecified dementia, and are often neglected within dementia care and research [48]. Yet, our understanding of the mortality burden of disease, which impacts both medical practice and the awarding of research grants to better understand the disease [55], is determined by the accuracy of the death certificate [56]. We are concerned that the 2014 coding rule change may be exacerbating the discrimination of people with Down syndrome by masking how severe the Alzheimer disease and unspecified dementia mortality burden truly is, which negatively impacts the needed attention to and funded research about dementia within this population [57].

In this study we examined the impact of the 2013 change to the selection rule for Alzheimer or unspecified dementia related deaths among people with Down syndrome. To do so, we document the rates of Alzheimer disease or unspecified dementia deaths and the leading causes of death among decedents with/without Down syndrome in the US from 2005–2019 using data as reported on the death certificate. We then contrast these findings with the actual burden of Alzheimer disease or unspecified dementia mortality after amending the death certificates reporting Down syndrome as the underlying cause of death. We expect to find that death certificates during and after 2013 likely report a lower percentage of Alzheimer or unspecified dementia deaths among those with Down syndrome than likely occurred. By doing so, we underscore the problem that the 2014 change presents to the study of Down syndrome mortality patterns.

## Methods

### Data and measures

This study uses 15 years (2005–2019) of the CDC’s National Center for Health Statistics’ (NCHS) National Vital Statistics System (NVSS) mortality data for adults aged 18 and over with (N = 27,911) and without Down syndrome (N = 38,396,633). Down syndrome was identified when ICD-10 code Q90.9 was reported anywhere on the death certificate.

We focus on the underlying cause of death (UCOD), the illness or injury reported to have started the chain of events leading to death. For adults without Down syndrome, we report the UCOD originally reported on the death certificate. For adults with Down syndrome, we report the UCOD originally reported, as well as an amended UCOD for adults who had Down syndrome reported as the UCOD. The amendment process relies on a sequential process to identify the amended cause of death [58–60]. For death certificates of adults with Down syndrome that did not report Down syndrome as the UCOD, the UCOD is not amended. For death certificates that did report Down syndrome as the UCOD, a valid UCOD was selected by working sequentially through the ICD-10 codes reported in Part I on the death certificate. The process proceeded from the last to first line of Part I of the death certificate, moving from the first to last listed code per line. ICD-10 codes that the CDC indicates should not be used as the UCOD were not permitted to be assigned as the amended UCOD [61]. ICD-10 codes from Chapter XVIII (commonly referred to as R-codes) were only used as the amended UCOD if no other valid UCOD was reported in Part I of the death certificate.

It is important to note that the reporting of Down syndrome as the UCOD is not exclusive to individuals who died from Alzheimer disease or unspecified dementia. In this study, of the 27,911 decedents with Down syndrome, 14,960 (53.6%) had Down syndrome reported as the UCOD. Only 4,364 of the 14,690 death certificates that had Down syndrome reported as the UCOD also had Alzheimer disease or unspecified dementia reported on the death certificate. Among these 4,364 cases that had Down syndrome reported as the UCOD with Alzheimer disease or unspecified dementia also reported on the death certificate, the amendment process only identified Alzheimer disease or unspecified dementia as the UCOD if it was: 1) listed sequentially before Down syndrome on the same line, or above Down syndrome on a distinct line; and 2) no other valid UCOD was reported between Down syndrome and Alzheimer disease or dementia. No single code received preferential consideration in amendment determinations. Of the 4,364 cases that had Down syndrome reported as the UCOD with Alzheimer disease or unspecified dementia also reported on the death certificate, 3,219 cases (73.8%) were amended to list Alzheimer disease or unspecified dementia as the UCOD. The other diseases most frequently identified as the UCOD by the amendment process for decedents who had Down syndrome reported as the UCOD with Alzheimer disease or unspecified dementia also reported on the death certificate were influenza/pneumonia (379 cases; 8.7%), heart disease (121 cases; 2.8%), and pneumonitis (104 cases; 2.4%).

As the 2013 change to reporting the UCOD for people with Down syndrome includes Alzheimer disease (ICD-10 code G30) and unspecified dementia (ICD-10 code F04), we focus on both for this study. Other UCODs used in the study are based on the CDC’s annual reporting of the leading causes of death in the US [8], inclusive of: Heart disease (ICD10 I00-I09, I11, I13, I20-I51); Cancer (C00-C97); Accidents (unintentional injuries) (V01-X59, Y85-Y86); Stroke (cerebrovascular diseases) (I60-I69); Chronic lower respiratory diseases (J40-J47); Diabetes (E10-E14); Influenza and pneumonia (J09-J18); Nephritis, nephrotic syndrome, and nephrosis (N00-N07, N17-N19, N25-N27); Septicemia (A40-A41); Chronic liver disease and cirrhosis (K70, K73-K74); Essential hypertension and hypertensive renal disease (I10, I12, I15); Parkinson disease (G20-G21); and Pneumonitis due to solids and liquids (J69).

### Analytic strategy

We begin by reporting the number and percentage of Alzheimer disease or unspecified dementia deaths overall and by year for adults with and without Down syndrome. We document these same trends separately for Alzheimer disease and unspecified dementia in the Appendices. We then report the leading causes of death for adults with and without Down syndrome for the entire study period, 2005–2019. When reporting the leading causes of death, we include data points for Alzheimer disease and unspecified dementia as a combined category, and as distinct categories. For adults with Down syndrome, analysis includes data points using the original and amended UCOD. Analysis was conducted with STATA 17.0 (College Station, TX).

## Results

### Number and percentage of deaths

As reported in Table 1, the overall percentage of deaths from Alzheimer disease or unspecified dementia were similar for adults with (8.0%) and without (7.4%) Down syndrome when the originally reported UCOD was examined for both groups. However, comparison with the amended UCOD for adults with Down syndrome revealed that this similarity was not an accurate reflection of the Alzheimer disease or unspecified dementia mortality burden for adults with Down syndrome. Instead, after completing the amendment process, the percentage of adults with Down syndrome who died from Alzheimer disease or unspecified dementia was 19.7%. While every year of data included cases that had Down syndrome reported as the UCOD when the decedent may in fact have died from Alzheimer disease or unspecified dementia, the number of such cases dramatically decreased starting in 2014. In fact, when examining the original UCOD for decedents with Down syndrome from 2015 through 2019, there was only 1 case in which Alzheimer disease or unspecified dementia was reported as the UCOD.

**Table 1:** Alzheimer disease or unspecified dementia death patterns among adults with/without Down syndrome, 2005–2019.

	No Down syndrome		Down syndrome			
	(N = 38,369,633)		(N = 27,911)			
	Original UCOD		Original UCOD		Amended UCOD	
	Number of deaths	Percentage of all deaths	Number of deaths	Percentage of all deaths	Number of deaths	Percentage of all deaths
2005–2019	2,850,518	7.43%	2,228	7.98%	5,493	19.68%
2005	123,371	5.13%	173	10.16%	270	15.86%
2006	137,421	5.77%	210	12.89%	238	14.61%
2007	146,676	6.16%	257	15.11%	286	16.81%
2008	165,514	6.81%	270	15.13%	297	16.65%
2009	163,665	6.83%	340	17.67%	362	18.81%
2010	181,414	7.46%	285	17.31%	323	19.62%
2011	196,627	7.93%	214	12.10%	355	20.07%
2012	205,855	8.21%	218	11.95%	383	21.00%
2013	213,806	8.35%	229	12.07%	377	19.86%
2014	215,556	8.32%	30	1.62%	373	20.10%
2015	218,465	8.16%	0	0.00%	420	21.68%
2016	217,036	8.01%	0	0.00%	432	21.08%
2017	223,422	8.03%	0	0.00%	440	20.66%
2018	222,635	7.93%	1	0.05%	450	22.31%
2019	219,055	7.76%	0	0.00%	487	23.81%

Comparison of the number and percentage of adults with and without Down syndrome (using the amended UCOD) who died from Alzheimer disease or unspecified dementia revealed an overall increase in the mortality burden of these diseases across the years of the study but with slightly different patterns. Among adults without Down syndrome there was a steady increase in the number of Alzheimer or unspecified dementia deaths from 123,371 in 2005 to 219,055 in 2019, with slight year-on-year decreases recorded in 2009, 2016, and 2019. Regarding percentage of deaths, there was an increase from 2005 to 2013, then a slight decrease from 2013 to 2019. Among adults with Down syndrome, the number of cases also increased steadily across years from 270 in 2005 to 487 in 2019, with slight year-on-year decreases recorded in 2006, 2010, 2013, and 2014. The percentage of deaths among adults without Down syndrome increased steadily from 2005 to 2012, plateaued through 2017, then slightly increased in 2018 and 2019. Similar patterns were observed when examining the same trends separately for Alzheimer disease (S1 Appendix) and unspecified dementia (S2 Appendix).

### Leading causes of death

The leading causes of death from 2005 to 2019 for adults with and without Down syndrome are reported in Table 2. For adults without Down syndrome, Alzheimer disease or unspecified dementia as a combined category was the 3^rd^ leading cause of death, accounting for 7.43% of all deaths. Considered as distinct categories, unspecified dementia (3.78%) was the 6^th^ leading cause of death and Alzheimer disease (3.65%) was the 7^th^ leading cause of death.

**Table 2:** Leading causes of death for adults with/without Down syndrome, 2005–2019.

No Down syndrome		Down syndrome			
(N = 38,369,633)		(N = 27,911)			
Original UCOD		Original UCOD		Amended UCOD	
Cause of death	%	Cause of death	%	Cause of death	%
Heart disease	24.42%	Down syndrome	53.60%	***Alzheimer disease/Unspecified dementia combined***	***19*.*68%***
Cancer	22.68%	Heart disease	8.58%	Heart disease	14.12%
***Alzheimer disease/Unspecified dementia combined***	***7*.*43%***	***Alzheimer disease/Unspecified dementia combined***	***7*.*98%***	Influenza and pneumonia	12.16%
Chronic lower respiratory diseases	5.65%	Pneumonitis due to solids and liquids	5.05%	***Alzheimer disease***	***10*.*00%***
Stroke (Cerebrovascular disease)	5.35%	***Alzheimer disease***	***5*.*00%***	***Unspecified dementia***	***9*.*68%***
Accidents (unintentional injuries)	5.16%	***Unspecified dementia***	***2*.*98%***	Pneumonitis due to solids and liquids	9.23%
***Unspecified dementia***	***3*.*78%***	Stroke (Cerebrovascular disease)	2.42%	Accidents (unintentional injuries)	3.36%
***Alzheimer disease***	***3*.*65%***	Accidents (unintentional injuries)	2.35%	Stroke (Cerebrovascular disease)	2.89%
Diabetes	2.98%	Cancer	2.27%	Cancer	2.29%
Influenza and pneumonia	2.13%	Septicemia	1.59%	Septicemia	2.26%
Nephritis, nephrotic syndrome and nephrosis	1.88%	Chronic lower respiratory diseases	1.16%	Chronic lower respiratory diseases	1.42%
Septicemia	1.45%	Diabetes	0.98%	Nephritis, nephrotic syndrome and nephrosis	1.39%
Chronic liver disease and cirrhosis	1.38%	Influenza and pneumonia	0.88%	Diabetes	1.23%
Essential hypertension and hypertensive renal disease	1.15%	Nephritis, nephrotic syndrome and nephrosis	0.69%	Parkinson disease	0.61%
Parkinson disease	0.99%	Parkinson disease	0.56%	Essential hypertension and hypertensive renal disease	0.44%
Pneumonitis due to solids and liquids	0.71%	Essential hypertension and hypertensive renal disease	0.28%	Chronic liver disease and cirrhosis	0.22%
		Chronic liver disease and cirrhosis	0.18%		

Highlighting the complexity of the coding rule change issue, when using the original UCOD, 53.6% of adults with Down syndrome had their Down syndrome reported as the UCOD. As a result, rates of death for all causes of death were lower compared to the amended UCOD. When examining the original UCOD reporting, Alzheimer disease or unspecified dementia was the 3^rd^ leading cause of death for adults with Down syndrome accounting for 7.98% of all deaths. As distinct categories, Alzheimer disease (5%) was the 4^th^ leading cause and unspecified dementia (2.98%) was the 5^th^ leading cause of death.

A very different story regarding the mortality burden of Alzheimer disease or unspecified dementia among adults with Down syndrome was apparent when using the amended UCOD to examine leading causes of death. This more accurate accounting of mortality burden demonstrates that when examined as a combined category, Alzheimer disease and unspecified dementia accounted for 19.68% of all deaths for adults with Down syndrome from 2005–2019, becoming the leading cause of death. When considered as separate categories, Alzheimer disease (10.00%) was the 3^rd^ leading cause of death, while unspecified dementia (9.68%) was the 4^th^ leading cause of death. The amended UCOD analysis had only modest effect on the rank order of other conditions, which were similar to those reported in a recent study focused on variation in causes of death among adults with/without Down syndrome [62].

## Discussion

Results from this study confirmed that there was a substantial shift in the overall percentage of deaths reported for adults with Down syndrome from Alzheimer disease or unspecified dementia after changes to coding rules were fully implemented in 2014. When not taking this change into consideration, it appeared that rates of death from Alzheimer disease or dementia between 2005 and 2019 were similar for adults with and without Down syndrome, and that Alzheimer disease and dementia was the 3^rd^ leading cause of death for both groups. Yet, this was not actually the case. Instead, after adjusting data to more accurately reflect the number of Alzheimer or unspecified dementia deaths among adults with Down syndrome: 1) the prevalence of Alzheimer disease or unspecified dementia deaths was 2.7 times higher for adults with than without Down syndrome; and 2) Alzheimer disease and unspecified dementia was the leading cause of death for adults with Down syndrome compared to the 3^rd^ leading cause for adults without Down syndrome. In addition, results demonstrate that rates of death from Alzheimer disease or dementia continued to increase over time for those adults with Down syndrome, while appearing to stabilize for adults without Down syndrome. This is a pattern that may be associated with a documented increase in age at death for adults with Down syndrome during this time period [50] and/or more attention to Alzheimer disease and unspecified dementia in people with Down syndrome among the medical community resulting in more awareness of this disease process by certifiers.

We feel that it is important to once again underscore that the reporting of Down syndrome as the underlying cause of death when death occurred from Alzheimer disease or unspecified dementia is not inaccurate per current CDC guidance. However, this study’s results clearly show that these changes are problematic for understanding the Alzheimer disease or unspecified dementia mortality burden for adults with Down syndrome. In effect, the change to the CDC coding rules regarding the reporting of Down syndrome in instances of death from Alzheimer disease or unspecified dementia produces a severe underestimation of the mortality burden of this disease among people with Down syndrome.

As seen in the annual report on Alzheimer disease published by the Alzheimer’s Association [14], information on rates of death from Alzheimer disease using underlying cause of death information is a key piece of evidence for understanding the burden of Alzheimer disease on the US population. Based on this vital information, the Alzheimer’s Association website emphasizes that “Alzheimer’s kills,” and that deaths from Alzheimer disease are increasing over time [63]. Clearly, accurate information on the rate of death from Alzheimer disease is critical to helping the public and medical providers understand the gravity of this disease. And yet, due to the changes in the CDC coding rules, the gravity of Alzheimer disease or unspecified dementia mortality burden is not readily apparent in death certificate data for adults with Down syndrome. This raises the practical question of whether the benefit of reporting Down syndrome as the underlying cause of death in instances when death actually occurred from Alzheimer disease or dementia outweighs having a more accurate understanding of mortality burden for this population. We fully understand that there appears to be a close association between Down syndrome and the development of Alzheimer disease. Yet, in light of the fact that doing so minimizes the actual burden of Alzheimer disease and unspecified dementia deaths among adults with Down syndrome, it is important to reconsider whether there is any justification for reporting Down syndrome as the cause of death when death actually occurred from Alzheimer disease or unspecified dementia.

Some researchers propose that analysis of multiple causes of death data provide a fuller picture of disease patterns than analysis of underlying cause of death data in general [64–67], and Alzheimer disease deaths in particular [68, 69]. Analyzing multiple cause of death data is useful when investigating comorbidities and change in health trends over time and offers a more comprehensive study of mortality including the potential identification of causal pathways [70, 71]. The strategy of focusing on multiple cause of death data, which provide information on disease-related mortality as opposed to disease mortality, may prove helpful when attempting to work around the 2013 CDC rule change regarding reporting Down syndrome in instances of death from Alzheimer disease or dementia. Doing so bypasses any concern with which of the conditions, Down syndrome or Alzheimer disease/unspecified dementia, is reported as the underlying cause of death, and can provide a better estimate of the number of decedents who died with or from dementia, reported as a multiple or underlying cause of death, as opposed to only from dementia, reported as the underlying cause. However, it is important to acknowledge multiple cause of death analysis is not without its own problems. A recent study by Iulita et al. [57] on Down syndrome and Alzheimer disease demonstrates that the high prevalence of Alzheimer disease among people with Down syndrome may provide a ceiling effect that limits life expectancy for this population [57]. They also show that Alzheimer disease among people with Down syndrome is likely under-reported on the death certificate [57]. Though not without limitations, and not directly addressing the concerns we highlight with the changes to the CDC coding rules on Down syndrome, we do think that examination of multiple cause of death data holds potential for better understanding comorbidity patterns among adults with Down syndrome who die with Alzheimer disease or unspecified dementia.

Limitations to this study center around concerns with the death certificate. In general, the accuracy of death certificate data is inherently bound by the lack of training on death certification among certifiers [23, 26], varying regulations on who is permitted to act as a death certifier [22, 28], and the reliance on the certifier’s best opinion of the decedent’s underlying cause of death [7, 20, 21]. This may result in differences in the accuracy and therefore the value these certificates provide in understanding mortality trends. Beyond general concerns with death certificate data, there is concern that those certifying death certificates may choose to report acute conditions, as opposed to the longer-term condition of Alzheimer disease or dementia, as the underlying cause of death [14]. In addition, it may be difficult for those certifying the death certificate to determine whether a death is from Alzheimer disease or unspecified dementia or just associated with these diseases [14]. For these reasons, it is likely that the number of deaths reported as due to Alzheimer or unspecified dementia are an under-count [14].

There are also concerns regarding the reporting of Down syndrome on the death certificate beyond those explored in this study. It is possible that in general there is an under-reporting of IDDs on death certificates. For this reason, it is imperative that medical providers receive more training regarding accurate reporting of these disabilities on death certificates. A recent paper provides thorough and clear recommendations on the accurate reporting of intellectual and developmental disabilities on death certificates [45]. However, it is important to understand that under-reporting of intellectual and developmental disabilities on death certificates is less common for more recognizable disabilities such as Down syndrome, than for other disabilities such as intellectual disability [72]. That appears to be the case in this study as prevalence of Down syndrome among adult decedents was 7.26 per 10,000, which is in the range of a recent estimate of the entire Down syndrome population in the US (8.27 per 10,000; 90% UI 6.14–10.62) [73] and slightly higher than the one estimate of adults with Down syndrome in the US (5.3 per 10,000; confidence interval not provided) [74]. Nonetheless, as under-reporting still may occur among decedents with Down syndrome, it is important to understand that results from this study regarding Down syndrome only represent decedents who had this disability status reported on their death certificate.

It is also important to point out that despite using a sequential process to amend the death certificates of those who had Down syndrome reported as the UCOD, this process can obviously not consider all factors present at the time of death. Based upon these limitations, we assume that the number of deaths from Alzheimer disease or unspecified dementia may be under-reported for adults with and without Down syndrome in general, and that the number of decedents with Down syndrome reported on their death certificate may also reflect an under-count of those who died with this disability during the course of the study.

## Conclusion

The use of vital registration data is necessary in order to understand the mortality patterns of a population. Health researchers typically use underlying cause of death in order to examine rates of death from specific conditions, as well as whether certain causes of death are more prevalent among one group of people than another. This study’s results demonstrate that a 2013 change to death certificate coding rules resulted in a severe under-reporting of the mortality burden from Alzheimer disease or unspecified dementia among people with Down syndrome.

We recognize that Alzheimer disease and unspecified dementia are closely linked among people with Down syndrome. But we think that the population health utility of the death certificate has been undermined by the 2013 decision to report Down syndrome as the underlying cause of death in instances when a person actually died from Alzheimer disease or dementia. Based on this study’s findings, this rule change has hidden the true burden of Alzheimer disease or unspecified dementia among people with Down syndrome. For this reason, we contend that the current rule instructing that Down syndrome be reported as the underlying cause of death in instances when the person actually dies from Alzheimer disease or unspecified dementia should be changed. We are concerned that not doing so will result in a misunderstanding of the severe impact of these diseases on people with Down syndrome, further marginalizing people with Down syndrome in dementia care needs and research efforts.

## Supporting information

S1 Appendix Alzheimer disease death patterns among adults with/without Down syndrome, 2005–2019. (DOCX)

S2 Appendix Unspecified dementia death patterns among adults with/without Down syndrome, 2005–2019. (DOCX)
