# Supplementary material for: Reporting Down syndrome on the death certificate for Alzheimer disease/unspecified dementia deaths
Source: PLoS One. 2023 Feb 13;18(2):e0281763. doi: 10.1371/journal.pone.0281763 (PMC9925077; doi:10.1371/journal.pone.0281763)
Supplement: S1 Appendix — (DOCX) [file pone.0281763.s001.docx]

Appendix 1: Alzheimer disease death patterns among adults with/without Down syndrome, 2005-2019

|  | No Down syndrome  N=38,369,633 | | Down syndrome  N=27,911 | | | |
| --- | --- | --- | --- | --- | --- | --- |
|  | UCOD | | Original UCOD | | Amended UCOD | |
|  | Number of deaths | Percentage  of all deaths | Number of deaths | Percentage  of all deaths | Number of deaths | Percentage  of all deaths |
| 2005-2019 | 1,401,048 | 3.65% | 1,396 | 5.00% | 2,790 | 10.00% |
|  |  |  |  |  |  |  |
| 2005 | 71,466 | 2.97% | 144 | 8.46% | 163 | 9.58% |
| 2006 | 72,330 | 3.04% | 116 | 7.12% | 128 | 7.86% |
| 2007 | 74,488 | 3.13% | 157 | 9.23% | 173 | 10.17% |
| 2008 | 82,310 | 3.39% | 142 | 7.96% | 149 | 8.35% |
| 2009 | 78,819 | 3.29% | 199 | 10.34% | 208 | 10.81% |
| 2010 | 83,348 | 3.43% | 162 | 9.84% | 172 | 10.45% |
| 2011 | 84,833 | 3.42% | 156 | 8.82% | 169 | 9.55% |
| 2012 | 83,501 | 3.33% | 149 | 8.17% | 158 | 8.66% |
| 2013 | 84,633 | 3.30% | 148 | 7.80% | 153 | 8.06% |
| 2014 | 93,545 | 3.61% | 22 | 1.19% | 144 | 7.76% |
| 2015 | 110,586 | 4.16% | 0 | 0.00% | 231 | 11.93% |
| 2016 | 116,137 | 4.29% | 0 | 0.00% | 224 | 10.93% |
| 2017 | 121,448 | 4.37% | 0 | 0.00% | 240 | 11.27% |
| 2018 | 122,065 | 4.35% | 1 | 0.05% | 223 | 11.06% |
| 2019 | 121,539 | 4.30% | 0 | 0.00% | 255 | 12.47% |
