# Supplementary material for: Reporting Down syndrome on the death certificate for Alzheimer disease/unspecified dementia deaths
Source: PLoS One. 2023 Feb 13;18(2):e0281763. doi: 10.1371/journal.pone.0281763 (PMC9925077; doi:10.1371/journal.pone.0281763)
Supplement: S2 Appendix — (DOCX) [file pone.0281763.s002.docx]

Appendix 2: Unspecified dementia death patterns among adults with/without Down syndrome, 2005-2019

|  | No Down syndrome  N=38,369,633 | | Down syndrome  N=27,911 | | | |
| --- | --- | --- | --- | --- | --- | --- |
|  | UCOD | | Original UCOD | | Revised UCOD | |
|  | Number of deaths | Percentage  of all deaths | Number of deaths | Percentage  of all deaths | Number of deaths | Percentage  of all deaths |
| 2005-2019 | 1,449,470 | 3.78% | 831 | 2.98% | 2,703 | 9.68% |
|  |  |  |  |  |  |  |
| 2005 | 51905 | 2.16% | 29 | 1.70% | 107 | 6.29% |
| 2006 | 65091 | 2.73% | 94 | 5.77% | 110 | 6.75% |
| 2007 | 72188 | 3.03% | 100 | 5.88% | 113 | 6.64% |
| 2008 | 83204 | 3.42% | 128 | 7.17% | 148 | 8.30% |
| 2009 | 84846 | 3.54% | 141 | 7.33% | 154 | 8.00% |
| 2010 | 98066 | 4.03% | 123 | 7.47% | 151 | 9.17% |
| 2011 | 111794 | 4.51% | 58 | 3.28% | 186 | 10.51% |
| 2012 | 122354 | 4.88% | 69 | 3.78% | 225 | 12.34% |
| 2013 | 129173 | 5.04% | 81 | 4.27% | 224 | 11.80% |
| 2014 | 122011 | 4.71% | 8 | 0.43% | 229 | 12.34% |
| 2015 | 107879 | 4.03% | 0 | 0.00% | 189 | 9.76% |
| 2016 | 100899 | 3.72% | 0 | 0.00% | 208 | 10.15% |
| 2017 | 101974 | 3.67% | 0 | 0.00% | 200 | 9.39% |
| 2018 | 100570 | 3.58% | 0 | 0.00% | 227 | 11.25% |
| 2019 | 97516 | 3.45% | 0 | 0.00% | 232 | 11.34% |
